# CT Findings of Undifferentiated Pancreatic Cancer With Osteoclast-Like Giant Cells: A Case Series from a Single Center's Experience With 10 Cases

**DOI:** 10.7759/cureus.43798

**Published:** 2023-08-20

**Authors:** Zhihong Lan, Weipeng Chen, Xiangrong Yu, Guofeng Zhou

**Affiliations:** 1 Department of Radiology, Zhuhai Hospital Affiliated with Jinan University, Zhuhai People’s Hospital, Zhuhai, CHN; 2 Department of Radiology, Zhongshan Hospital, Fudan University, Shanghai, CHN

**Keywords:** computed tomography (ct), case series, diagnosis, pancreas, undifferentiated pancreatic cancer with osteoclast like giant cells

## Abstract

Little is known about the imaging features of undifferentiated carcinoma with osteoclast-like giant cells of the pancreas (UCOGCP) because of its extremely low incidence. To improve the diagnostic accuracy of this tumor, 10 UCOGCP cases with confirmed histopathology were collected and their clinical and image data features were analyzed. We found that the median age of our study was 61 years (50-76 years in range) and the main clinical manifestations were nonspecific abdominal pain. There were some differences in the degree of enhancement and computed tomography (CT) features between the tumor located at the head and body or tail of the pancreas. Perhaps these subtle imaging findings can provide valuable diagnostic information.

## Introduction

Pancreatic giant cell tumor (PGCT) is a rare non-endocrine tumor of the pancreas. It occurs in less than 1% of all pancreatic tumors [1] and in 2% to 7% of all pancreatic cancers [2]. It was originally described by Juan Rosai in 1968 as a variant of undifferentiated carcinoma [1]. There are three types of PGCT: osteoclastic, pleomorphic, and mixed type [3]. It was defined as undifferentiated carcinoma with osteoclast-like giant cells of the pancreas (UCOGCP), which was a rare variant of pancreatic ductal carcinoma (PDC) until the 4th edition of WHO 2010 digestive tract tumors. If the tumor is found at an advanced stage and cannot be completely removed, the prognosis is extremely poor [4], with an average survival of less than a year [5]. However, little is known about the imaging characteristics of UCOGCP. In this article, we will review and analyze computed tomography (CT) imaging of 10 previously pathologically confirmed cases in search of distinct or suggestive image signatures. We follow recently developed AME Case Series Checklist guidelines to optimize the quality of reporting of clinical findings in cases.

## Materials and methods

Ethics committee and written informed consent exemption were obtained in this retrospective study. Ten cases with surgically and histologically proven UCGOCP were selected for this retrospective study by reviewing documented abdominal CT examinations from September 2014 to December 2018 in the radiology database of Zhongshan Hospital, Fudan University. All patients underwent spiral CT enhanced scan (GE Light Speed 64-slice spiral CT) pre-operation. The imaging parameters of all phases were as follows: 120 kV tube voltage, 1.25 mm thickness, automatic dose modulation of 90-270 mA tube current, and 256×256 matrix 1.5ml/kg nonionic iodinated contrast agent (Optiray 320, TycoHealthcare, Quebec, Canada) was administered intravenously through the catheter at a rate of 3-4 milliliters per second. The arterial phase image was obtained after the injection tracking program detected a descending aorta threshold of 200 Hounsfield units (HU) and a 5-second delay. Subsequently, venous phase images were acquired around 60 seconds later.

Two senior radiologists evaluated the following CT features in consensus: tumor location, maximum diameter (the largest tumor diameter on three-dimensional reconstructions), homogeneity, margin delineation, calcification, contrast enhancement, tumor-to-pancreas contrast ratio, pancreatic duct dilatation, vascular involvement, pancreatic atrophy, and liver metastases.

Oval regions of interest (ROI) of 5mm^2^ were set at the same place of the tumor avoiding cyst, calcification, and vessel, to measure the density of the tumor of each phase. The dominant part of the enhancement is measured in the presence of tumor heterogeneity. The relative tumor enhancement rate, defined as the value (Hu) of tumor density divided by the normal pancreatic parenchyma, was measured at both the arterial and venous stages. Hematoxylin and eosin (H&E) stain and histopathologic examination were also performed on operative specimens.

## Results

Ten patients' clinical characteristics are summarized in Table 1. The median age of the patients was 61 years. Cases were slightly more frequent in males (n = 6). The most common presentation was abdominal pain in eight cases, lasting from two weeks to two months and one case had the clinical manifestation of jaundice at the same time. Additional symptoms included abdominal distention (n = 2), backache (n = 2), and melena (n = 1). One patient was found due to a physical examination. At admission, in four patients carbohydrate antigen 19-9 (CA19-9) was confirmed to be elevated. Three patients underwent distal pancreatectomy with splenectomy and seven underwent pancreaticoduodenectomy during the procedure.

**Table 1:** Clinical findings in 10 patients with UCOGCP M, male; F, female; age: year; symptom duration: lesions were found on physical examination and the course of the disease was unclear; CA19-9; tumor markers whose normal reference value was less than 27 u/ml. UCOGCP: undifferentiated carcinoma with osteoclast-like giant cells of the pancreas; CA19-9: carbohydrate antigen 19-9

Case/sex/age	Clinical presentation	Symptom duration	CA19-9 (u/ml)
1/F/76	Abdominal pain with fever	2 weeks	48.9
2/M/53	Lower abdominal pain and backache	2 weeks	38.6
3/M/57	Abdominal distension and jaundice	2 weeks	Normal
4/M/68	Normal	-	73.7
5/F/65	Bilateral backache	1 year	Normal
6/M/60	Abdominal distension	10 days	Normal
7/F/53	Abdominal pain	2 months	Normal
8/F/73	Abdominal pain	1 month	63.7
9/M/55	Melena	-	Normal
10/M/50	Abdominal pain	2 weeks	Normal

**Figure 1:**
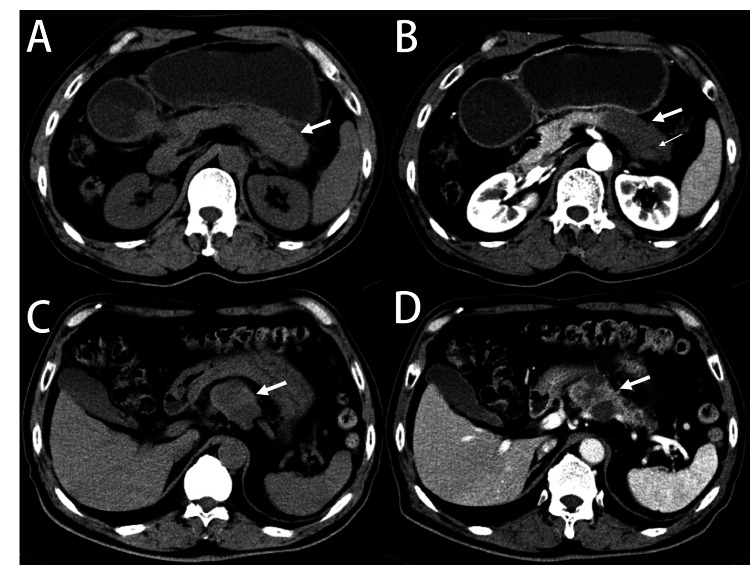
Undifferentiated carcinoma with osteoclast-like giant cells appeared as cystic and solid lesions. (A) A 60-year-old man with undifferentiated carcinoma with osteoclast-like giant cells in the tail of the pancreas. The volume of a pancreatic tail was increased (white arrow). (B) An enhanced CT scan showed a major solid lesion with hypo enhancement (thick arrows) and a small cystic lesion (thin arrows) was visible. (C) Another 76-year-old man with undifferentiated carcinoma with osteoclast-like giant cells in the tail of the pancreas. Mixed-density lesions (white arrows) are seen in the tail of the pancreas. (D) An enhanced CT scan showed that the major cystic lesions were not enhanced, and the solid nodule at the edge of the lesion was enhanced. CT: computed tomography

**Figure 2:**
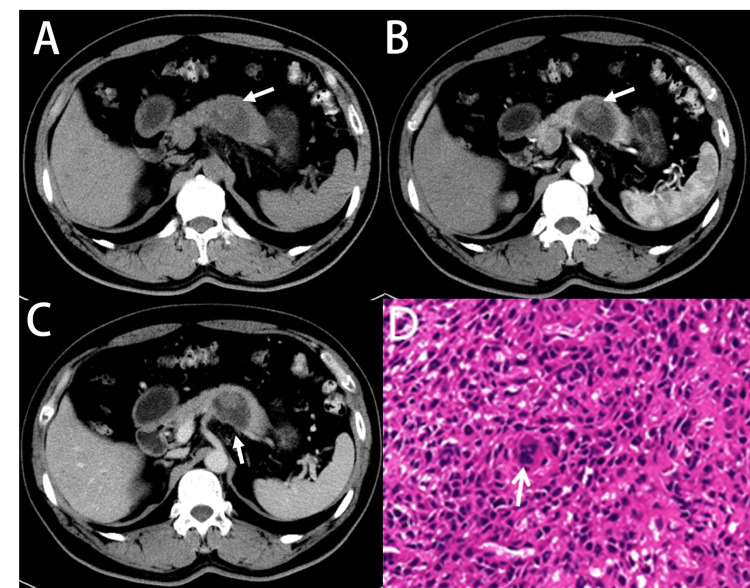
A 53-year-old man with undifferentiated carcinoma with osteoclast-like giant cells in the pancreatic body. Axial CT showed a well-defined predominantly cystic lesion. On non-enhanced CT, the density of the lesion (white arrow) is lower than that of normal pancreatic tissue, and its CT value is 27 HU. (A) In arterial lesions (white arrows), the enhancement attenuation is very low (42 HU), and cystic necrosis was seen in the lesion, peripheral nodule giving a “marginal island” like appearance. (B) The superior mesenteric artery (white arrow) is infiltrated, but there was no lymph node metastasis. (C) Histological examination revealed undifferentiated cancer, which was a mixture of neoplastic polymorphic monocytes and abundant non-neoplastic osteoclast-like giant cells and histiocytes (D, white arrow). CT: computed tomography

The CT characteristics of UCOGCP were summarized (Table 2 and Table 3). All cases were irregular masses (the median longest dimension was 43 mm). The tumor can occur in any part of the pancreas, including the uncinate process (2/10), head near the duodenum (2/10), neck (2/10), body (2/10), and tail (2/10). A single lesion was found in all cases. Cystic regions were visible in each case. There was no calcification in the tumor, and the margin of the tumor was well-circumscribed. No atrophy of the pancreas was seen in our study. In the unenhanced period, the CT value (mean±SD) of the tumor was 37.5±6.8 HU, which showed iso- or slightly hyper-density to the pancreatic parenchyma. After contrast injection, the tumor showed heterogeneous enhancement. The lesions’ CT value was 64.2±16.1 HU during the arterial phase, and 71.5±16.5 HU during the venous phase, which showed low attenuation relative to pancreatic parenchyma. Most lesions appeared as predominantly cystic lesions (n = 6) (Figures 1A-1B), and the rest appeared as predominantly solid lesions (n = 4) (Figures 1C-1D). Half of the patients (5/10) had vascular involvement (Figure 2C). Lymph node metastases and distant metastases were not observed in any of our patients.

**Table 2:** CT findings in 10 patients with UCOGCP M, male; F, female; age: year UCOGCP: undifferentiated carcinoma with osteoclast-like giant cells of the pancreas; CT: computed tomography

Case/sex/age	Maximum diameter (mm)	Location	Tumor pattern	Margin	Calcification	Pancreatic duct dilatation	Vascular involvement
1/F/76	66	Tail	Predominantly cystic lesions	Well-circumscribed	-	-	+
2/M/53	38	Body	Predominantly cystic lesions	Well-circumscribed	-	-	+
3/M/57	24	Head	Predominantly cystic lesions	Well-circumscribed	-	+	-
4/M/68	44	Body	Predominantly cystic lesions	Well-circumscribed	-	-	-
5/F/65	19	Uncinate process	Predominantly solid lesions	Well-circumscribed	-	-	+
6/M/60	60	Tail	Predominantly solid lesions	Well-circumscribed	-	-	+
7/F/53	31	Uncinate process	Predominantly solid lesions	Well-circumscribed	-	-	+
8/F/73	59	Head	Predominantly cystic lesions	Well-circumscribed	-	-	-
9/M/55	42	Head (near duodenum)	Predominantly cystic lesions	Well-circumscribed	-	+	-
10/M/50	81	Head (near duodenum)	Predominantly solid lesions	Well-circumscribed	-	+	-

**Table 3:** The CT values of the solid part of the tumor on the unenhanced phase, arterial phase, venous phase, and tumor-to-pancreas contrast ratio images of UCOGCP M, male; F, female; age: year; SD: standard deviation UCOGCP: undifferentiated carcinoma with osteoclast-like giant cells of the pancreas; CT: computed tomography

Case/sex/age	Unenhanced phase	Arterial phase	Venous phase	Tumor-to-pancreas contrast ratio	
				Arterial phase	Venous phase
1/F/76	35HU	53HU	61HU	0.5	0.6
2/M/53	27HU	42HU	43HU	0.4	0.5
3/M/57	44HU	59HU	68HU	0.7	0.8
4/M/68	48HU	63HU	80HU	0.6	0.7
5/F/65	45HU	67HU	70HU	0.8	0.9
6/M/60	39HU	41HU	52HU	0.4	0.5
7/F/53	31HU	90HU	97HU	0.9	0.8
8/F/73	35HU	70HU	90HU	0.9	0.8
9/M/55	39HU	82HU	74HU	1.0	0.9
10/M/50	32HU	75HU	80HU	0.8	1.0
mean±SD	37.5±6.8HU	64.2±16.1HU	75.1±16.5HU	0.76±0.2	0.79±1.6

**Figure 3:**
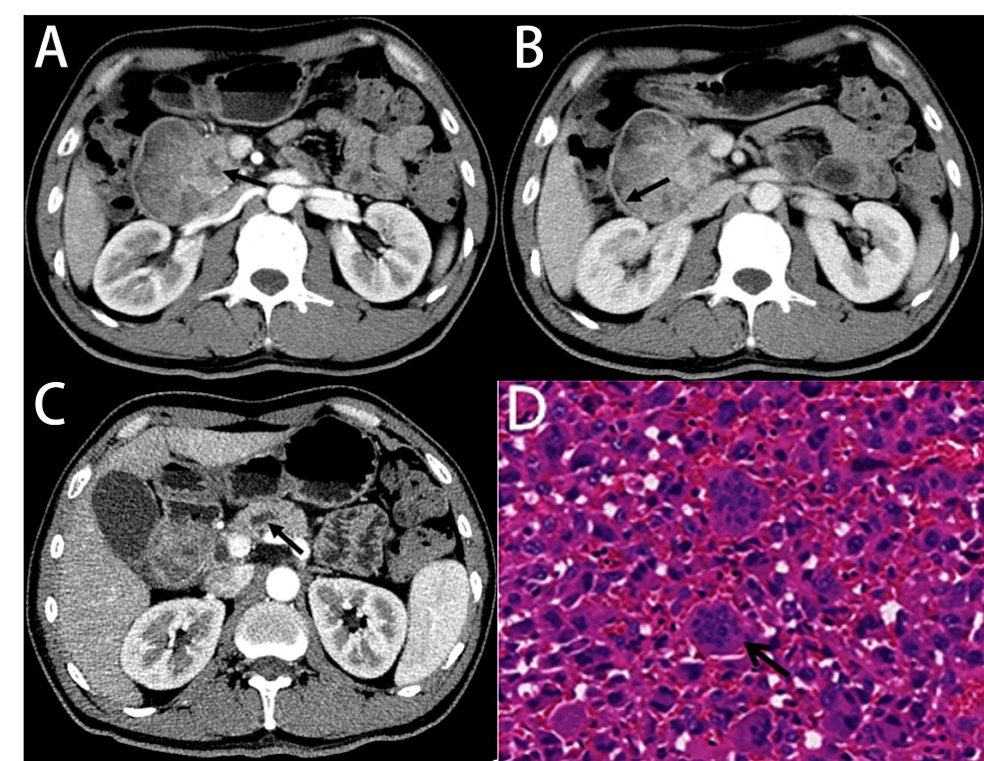
A 50-year-old patient with undifferentiated carcinoma with osteoclast-like giant cells in the pancreatic head. CT showed that the head tumor directly extended into the adjacent duodenum, which forming a “cauliflower-like” mass. Relative to the pancreatic parenchyma, the lesions showed a lower attenuation during the arterial phase (75 HU) (A, black arrow). During the venous phase (80 HU), the margin of the lesions became clear and smooth (B, black arrow). There is upstream pancreatic duct (black arrow)/common bile duct dilatation without pancreatic atrophy (C). Histological examination revealed undifferentiated cancer, which was a mixture of neoplastic polymorphic monocytes and abundant non-neoplastic osteoclast-like giant cells and histiocytes (D, black arrow). CT: computed tomography

Combining the biological behavior characteristics and imaging characteristics of tumors, we divided the cases into two groups for further observation. One group was the lesion that occurred in the pancreatic head (6/10), their enhancement degree of these lesions was found to be increased, with the tumor-to-pancreas contrast ratio reaching 0.8-1.0 (Table 3). Especially if the tumor was located near the duodenum (2/6), it will have special imaging features. There was no jaundice in the clinical presentation. The principal part of the tumor was located in the duodenum through the sphincter of Oddi, forming a “cauliflower-like” mass. The tumor had a maximum diameter of 81 mm, and the duct upstream of the pancreas was dilated. However, the pancreatic ducts were unobstructed (Figures 3A-3C). The other group was in a more common situation, in which the lesions were not located near the duodenum. Such lesions also had certain imaging signatures. Most of the cases in this group were predominantly cystic lesions. Moreover, some lesions were necrotic and only had peripheral solid nodules, giving a “marginal island-like appearance” (Figures 2A-2B). The tumor-to-pancreas contrast ratio of this group was always less than 0.8 (Table 3).

Histological examination revealed undifferentiated cancer, which was a mixture of neoplastic polymorphic monocytes and abundant non-neoplastic osteoclast-like giant cells and histiocytes. Non-tumorous osteoclast-like giant cells can be found in all cases (Figures 2-3).

## Discussion

UCOGCP is more common among older adults, 94% of whom are 50 or older, with an average age of 63 [6]. No obvious gender tendency had been reported [7]. The main clinical manifestations were nonspecific abdominal pain, abdominal distention, and obvious mass [8,9]. Our findings were similar to those that have been well documented, in which the median age of these 10 patients was 61 years and the most common presentation was abdominal pain.

Due to the rarity of UCOGCP, it is difficult to make a diagnosis based on its clinical features. Unlike common malignancies, we find that the margins of all tumors are always well-circumscribed. There were several previous case reports that described the imaging performance, where UCOGCP was mostly found in the body or tail of the pancreas with an incidence of about 70% [6]. However, in our cases, less than half of the patients had lesions located in the body and tail of the pancreas (4/10), which is inconsistent with literature reports. Several studies have reported that UCOGCP was generally large with a mean diameter of 8 cm [10], and the largest UCOGCP reported was 24.5 cm [9]. The literature also reported that the tumor was more common with hemorrhagic necrosis and marginal calcification [11]. However, in our case study, though cystic disease changes were common, the tumors were small, with the longest size ranging from about 19 to 81 mm (median 43 mm) but without any calcification. We speculated that the inconsistent results may caused by the tumor's discovered time being relatively earlier, where the time to form necrosis was short, but calcification was long.

In our study, cases were divided into two groups based on biological behavior and tumor location. When the tumor is located on the head of the pancreas, the degree of focal enhancement is significantly increased, approaching the density of the adjacent normal pancreas parenchyma, as the focal point tends to have a relatively abundant vascular network. In addition, the tumor that occurs on the pancreatic head near the duodenum can grow into the duodenal cavity through the sphincter of Oddi, forming a “cauliflower-like” mass. In this condition, both cases had slight dilatation of the upper pancreatic duct on CT images. It has been reported that since osteoclast-like giant cells in UCOGCP are composed of benign giant cells in the background of infiltrating anaplastic mononuclear malignant cells, they can fill and replace ducts in the pancreas [4]. The enhancement degree is significantly lower than the density of adjacent normal pancreatic parenchyma in another group where the tumor occurs on the body or tail. Tumor-to-pancreas contrast ratio is always <0.8. In addition, some tumors can form peripheral nodules, the rest are necrotic cysts, similar to marginal islands-like (Figure 2C). All of these tumors have progressively enhanced scans in our cases, which was similar to YHSHIHIKO F finding [12]. We also observed the central cystic mass with necrosis on the tumors’ images. It is the same as previous research findings [7] which may indicate that the tumor growth may be too fast to keep up with the blood supply.

Although the tumor size in our study was large, we did not find any tissue infiltration or lymph node metastasis. Remote metastasis can occur in the late stage, usually involving the liver, lungs, and bones [4]. Muraki et al. [5] found that lymph node metastasis in UCOGCP was less as compared to PDC. When the tumor is in late progression, distant metastases were present in 23% of cases of UCOGCP compared to 64% in PDC (p < 0.0001) [4]. Our research findings are consistent with the results reported in the literature, with no metastasis. En-bloc resection is commonly the first-line treatment. UCOGCP has a more favorable prognosis with a five-year survival rate of 59.1% compared to 15.6% for PDC [5].

The histological characteristics of UCOGCP were different from those of common PDC. For example, UCOGCP includes extremely pleomorphic tumor cells and large multinucleated non-neoplastic osteoclast-like giant cells that may develop from bone marrow-derived monocytes recruited into the tumor by chemical attractants [13]. The presence of non-neoplastic osteoclast-like giant cells is the histological hallmark of this tumor and the diagnosis is confirmed by histopathological and immunohistochemical studies [14]. ​Its diagnosis was confirmed by a positive result for the tissue monocyte marker CD68. The detection of Ki-67 may indicate a lack of mitotic and proliferative activity [4]. Our histological findings are consistent with the literature. Non-tumor osteoclast-like giant cells can be found in all cases. However, the time span of case collection was so great that most cases did not undergo immunohistochemistry.

​UCOGCP is often misdiagnosed as PDC prior to surgery due to its rarity and lack of understanding of imaging features. In pancreatic cancer, 90% of pathological manifestations are PDC, which, like giant cell carcinoma, originates in ductal cells. Although they are both tumors with poor blood supply, cystic degeneration and necrosis are rare in PDC. Moreover, PDC is characterized by peri-nerve infiltration and neurotrophic growth, accompanied by pancreatic atrophy and pancreatic duct dilatation [12], while UCOGCP's pancreatic duct dilation is not accompanied by pancreatic atrophy. Additionally, typical PDCs have ill-defined irregular tumor edges and are almost locally invasive, while UCOGCP appears as well-defined smooth tumor edges. UCOGCP also needs to be distinguished from other pancreatic solid cystic masses, such as solid pseudopapillary neoplasm (SPN), but SPN is more common in young women and does not have pancreatic duct dilatation [15].

Here are some limitations of our research. One is that the disease is so rare that we can collect few cases. Due to the large time span of collection, some cases were not followed up, and our pathological examinations had not been performed immunohistochemically.

## Conclusions

In conclusion, despite the rarity of UCGOCP, we can still find some imaging signatures for diagnosis. Most lesions are mainly cystic, with well-defined edges, and are accompanied by hemorrhagic necrosis. When the tumor is on the head of the pancreas, the enhancement of the lesion is close to that of a normal pancreas, especially if located near the duodenum, the cauliflower-like feature and the expansion of the pancreatic duct without the atrophy of the pancreas may have a cueing function. When tumors occur elsewhere in the pancreas, although the enhancement of the lesion is lower than in the normal pancreas, similar to PDC, the features of marginal vasodilation, central cyst with necrosis, and marginal island-like features are helpful for differential diagnosis.
